# Quantitative Risk Assessment of *Listeria monocytogenes* in Foods

**DOI:** 10.3390/foods14193353

**Published:** 2025-09-27

**Authors:** Juliana De Oliveira Mota, Michel Federighi

**Affiliations:** 1 Department of Nutrition and Food Safety, World Health Organization, 1211 Geneva, Switzerland; 2 Laboratoire de Sécurité des Aliments, Anses, 94700 Maisons-Alfort, France; 3 HQSA Unit, Ecole Nationale Vétérinaire d’Alfort, 94700 Maisons-Alfort, France

## 1. Introduction

Listeriosis is a rare foodborne infection caused by *Listeria monocytogenes*, with 0.1 to 10 cases per 1 million people per year according to the World Health Organization [1]. It is a 1–2 µm long bacillus that can multiply at low temperatures and in the presence of high salt concentrations. This bacterium is ubiquitous: it can be found in water, soil, plants, and many animals. In addition, this bacterium has the ability to contaminate ready-to-eat foods and food production sites. It is sensitive to heat but can multiply slowly at low temperatures.

Often occurring sporadically, their widespread dissemination in a wide range of foods can cause epidemics. It is important to remember that vertical transmission (from mother to fetus) through the placenta also occurs. The main clinical forms of listeriosis include septicemia and infection of the central nervous system or neuro-listeriosis, associated with a mortality rate of 30 to 45%, respectively, and maternal–neonatal forms. For example, in France, there is a ratio of 10 between neonatal forms (40/year) and other forms (400 to 500/year).

The scope of this Special Issue ‘Quantitative Risk Assessment of *Listeria monocytogenes* in Foods’ is twofold: (1) it aims to present a critical analysis of the existing human listeriosis quantitative risk assessment models conducted on milk and dairy products, produce, seafood, and meat products, highlighting common results from intervention strategies despite the unavoidable differences in model scopes and assumptions; (2) it also aims to present new quantitative risk assessment models of a longer food chain scope, in the light of new data and approaches, to control listeriosis linked to specific foods such as frozen vegetables, cantaloupe, and ready-to-eat seafood. In response to a request by the Codex Committee on Food Hygiene (CCFH) during its 52nd session, formal risk assessment models were developed by the Joint FAO/WHO Expert Meeting on Microbiological Risk Assessment of *Listeria monocytogenes* in Foods: Part 1 [2], taking into account the effects of agrifood practices, climate change, and the latent possibility of cross-contamination along the food chain. Programmed models were subsequently tested and revised at the Joint FAO/WHO Expert Meeting on Microbiological Risk Assessment of *Listeria monocytogenes* in Foods, Part 2: Risk Assessment Models [3].

## 2. Overview of the Published Articles

### 2.1. Critical Reviews of Risk Assessment Models for Listeria monocytogenes in Foods

This Special Issue provided the opportunity to critically review existing risk assessment models for four food commodities groups, namely dairy products [4], meat and meat products [5], seafood [6], and produce [7]. The reviews aimed to identify and evaluate the relative effectiveness of the control measures and intervention strategies along the food chain. 

The first review focused on dairy products [4]. Eighteen quantitative risk assessment (QRA) models were identified with a focus on cheeses, especially those made from raw and pasteurized milk, often within long supply chains. A key finding was the important role of the on-farm contamination in overall listeriosis risk, particularly in the context of raw milk cheeses. Consumer handling practices had greater influence on listeriosis risk than retail conditions, while storage temperature was found to have a higher impact than the storage time. 

The review of the existing QRA for *L. monocytogenes* in meat and meat products identified 23 models with a main focus on ready-to-eat (RTE) meat products and short supply chains [5]. Key factors influencing risk estimations included lag time, growth rate, and maximum microbial density, especially under the influence of growth inhibitors and lactic acid bacteria. Retail conditions emerged as an important contributing factor to risk, with deli meat sliced during retail presenting higher potential risks than pre-packed products. As for dairy, storage temperature was shown to have more impact on risk than storage duration. However, reducing *L. monocytogenes* prevalence and levels at the end of processing was found to be more effective than relying on storage conditions later in the chain, highlighting the need to incorporate processing modules into risk models.

For seafood [6], most of the 13 models focused on smoked or graved fish, all with short scopes, from the end of processing (packed products) to consumption, thus excluding cross-contamination modeling. As with previous food commodities, controlling storage temperature was more effective than acting on the storage duration. Again, prevalence and levels at the end of processing were found to be more effective than relying on storage conditions later in the chain. Therefore, this highlighted the importance of incorporating processing modules in order to better control the levels of microbial contamination at an earlier stage.

Finally, a review of produce identified 13 relevant articles, most of which focused on fresh or RTE leafy greens [7]. None of the models accounted for key factors or sources of contamination during primary production, such as the type of cultivation, water, fertilizers, or irrigation method/practices. Cross-contamination during processing was modeled using transfer rates, which moderately influenced the final risk, emphasizing the importance of accurately representing the transfer coefficient parameters. Temperature fluctuations at retail and temperature misuse during home storage were consistently identified as major contributors to listeriosis risk. The review advocated for extending the scope to include a primary module to assess current on-farm practices and controls, as well as to incorporate a refined sanitation module to test their effectiveness. 

Across all food categories, the review concluded that the enhanced integration of cross-contamination pathways and emerging technologies, the incorporation of detailed processing modules, and the use of well-informed kinetic parameters, such as the exponential growth rate, minimum growth temperature, and maximum population density, are essential steps toward more robust and realistic QRA models for *L. monocytogenes*.

### 2.2. Updated Dose–Response Models 

Pouillot et al. [8] developed the updated dose–response (DR) model incorporating both the virulence class of the *L. monocytogenes* strain and demographic factors such as age and sex. This model improves the accuracy of risk estimation across population groups and strain types, providing the necessary data are available. An open-source R package was developed to facilitate the practical application of these DR models. 

### 2.3. Quantitative Risk Assessment Models for Listeria monocytogenes

Building on the previous critical reviews and expert inputs, QRA models for *L. monocytogenes* were developed in order to estimate the risk of invasive listeriosis associated with the consumption of three different food commodities, namely non-RTE frozen vegetables [9], RTE smoked and graved fish [10], and RTE cantaloupe melon [11].

Gonzales-Barron et al. [9] developed the QRA model for non-RTE frozen vegetables, beginning at the freezing facility, with the assumption that incoming vegetables were already preconditioned (e.g., trimmed, peeled, and washed). The model incorporates the effect of blanching, potential cross-contamination during freezing and packaging, and consumer handling activities such as defrosting and cooking. 

Gonzales-Barron et al. [10] described a QRA model for RTE cold-smoked salmon produced by brining. The model covered other production steps such as smoking, slicing, and packing. It also accounted for cold-chain storage and consumer handling.

The last model developed by Guillier et al. [11] aimed to estimate the risk of listeriosis from the consumption of RTE cantaloupe. The model includes stages from pre-harvesting, harvesting, cleaning, and washing to processing, cold-chain storage, and consumer handling. 

Stochastic modeling was applied to simulate the risk of listeriosis, accounting for both within-lot variability (differences between individual units in a single production lot) and between-lot variability (differences across production lots). The models also simulated within-lot end-product testing scenarios. 

Risk is first estimated for each unit and then averaged across the units to produce a lot-level risk. Subsequently, the average or median risk per serving is calculated across all lots, providing an overall estimate used for comparison and scenario analysis.

The QRA model was made freely available as an open-source R package with full documentation and can be used as a tool to inform the consideration of strengthened risk management measures in view of the current changes in consumer behavior and new diet trends.

## 3. Conclusions

The models developed in this Special Issue are valuable tools for health authorities and food business operators to make the necessary decisions regarding this important public health issue. These tools are particularly useful and, moreover, are scalable. Nevertheless, they face certain biological limitations inherent to the specific microorganism *Listeria monocytogenes*.

In particular, they do not take into account this microorganism’s ability to colonize various abiotic surfaces by forming biofilms, particularly in industrial food environments. The genomic diversity of *L. monocytogenes* is significant and is linked to this ability to form biofilms for certain strains but also to different levels of virulence (hyper- or hypovirulent strains) [12]. There is no doubt that incorporating this data into models will improve the accuracy of quantitative risk assessments. In addition, it is known that this microorganism is capable of adopting viable but non-culturable forms, which are undetectable by standard research methods and can lead to risk underestimation [13].

This Special Issue highlighted the potential variations in risks associated with *L. monocytogenes* under different agricultural and processing conditions. The main results also highlight the importance of maintaining good agricultural practices in the field, adhering to strict hygiene practices during harvesting and processing, and ensuring appropriate storage conditions and handling practices by consumers in order to mitigate the risk of listeriosis.

At the same time, phylodynamic studies are needed to better understand the plasticity of the *Listeria* genome, which leads to the coexistence of several subtypes of *Listeria monocytogenes* in the same reservoir [14]. We also need to better understand the relationship between the improved fitness of *L. monocytogenes* and its ability to spread in many biotopes and/or cause more disease [15]. This “old” pathogen certainly continues to surprise us, and there is still much to learn about it.

## Data Availability

Not applicable.
